# Data on the effect of structure, elemental and phase composition gradient of nitride multilayer coatings on corrosion protection of different substrates in 3% NaCl and 5% NaOH solutions

**DOI:** 10.1016/j.dib.2019.104796

**Published:** 2019-11-11

**Authors:** Anna Kameneva, Vladimir Kichigin, Nikolay Lobov, Natalia Kameneva

**Affiliations:** a Perm National Research Polytechnic University, Perm, Russian Federation; b Perm State University, Perm, Russian Federation

**Keywords:** Single layer nitride coatings, Phase and elemental composition, Structure and composition gradient, Corrosion properties, Solutions of 5% NaOH and 3% NaCl

## Abstract

The single-layer nitride coatings (TiN; ZrN; (Ti,Zr)N; (Ti,Al)N; (Ti,Zr,Al)N) were deposited by DC magnetron sputtering (MS), cathodic arc evaporation (CAE), pulsed magnetron sputtering (PMS), and combined methods (CAE + MS and CAE + PMS). During the single-layer coating deposition period, only one of the technological parameters was changed: evaporator arc current (I_arc_ = 75–80 A), magnetron discharge power (N = 1.5–9.0 kW), bias voltage on the substrate (U_bias_ = 40–200 V), partial pressure of the gas mixture of nitrogen and argon (P = 0.24–1.4 Pa), the nitrogen content in the gas mixture (N_2_ = 12–100%) and the duration of the coating deposition (T_c_ = 5–45 min). The single-layer nanostructured coatings with preset structure, elemental and phase composition were obtained after optimization of deposition technological parameters. The failure pattern and surface morphology, grain size and coating thickness of single-layer and multi-layer coatings were examined using the field emission electron microscope Ultra 55 with EDAX microanalyzer. To determine metal concentration in the coatings, a local chemical analysis was carried out by EDAX microanalyzer. The corrosion behavior of the single-layer two-, three- and multicomponent nitride coatings on the hard alloy, tool steel, and low carbon steel in the 5% NaOH and 3% NaCl was evaluated employing electrochemical techniques such as electrochemical impedance spectroscopy (EIS) and polarization curves. Multilayer nitride coatings were developed on the basis of single-layer coatings with a maximum protective effect in the 5% NaOH and 3% NaCl. The data of phase and elemental composition, and corrosion properties of single layer nitride coatings was presented in detail.

**Table:** Specifications Table

Subject area	*Surfaces, Coatings and Films*
More specific subject area	*Coating structure, phase and elemental composition, corrosion protection, PVD method*
Type of data	*Table, image (microscopy), text file, graph, figure*
How data was acquired	*The hard alloy VK8 (HG30 – standard DIN), tool steel X12 M (D2 – ASTM or X165CrMoV12 – DIN), and low carbon steel St3 (1017 – ASTM and USt 37-2 – DIN) were used as substrates.* *The elemental composition of the magnetron's target and evaporator's cathode is as follows: titanium VT1-00 (Ti – 99.5–99.9%; Fe – up to 0.12%; C – up to 0.05%; S – up to 0.08%; N*_*2*_*– up to 0.04%; O – up to 0.1%), and aluminum alloy A85 (Al - 99.8%; Fe - up to 0.08%; Si - up to 0.06%; Ti up to 0.01%; Cu up to 0.01%; Zn up to 0.02%).* *The single-layer coatings were deposited by DC magnetron sputtering (MS), cathodic arc evaporation (CAE), pulsed magnetron sputtering (PMS), and combined methods (CAE + MS and CAE + PMS). Deposition was carried out at varying values of one of the technological parameters: evaporator arc current (I*_*arc*_ = *75–80 A), magnetron discharge power (N* = *1.5-9.0 kW), bias voltage on the substrate (Ubias* = *40–200 V), partial pressure of the gas mixture of nitrogen and argon (P* = *0.24-1.4 Pa), the nitrogen content in the gas mixture (N2 = 12–100%) and the duration of the coating deposition (Tc* = *5–45 min). The multilayer coatings included layers deposited at optimum values of the technological parameters. The data of phase and elemental composition, structure, and corrosion properties of single layer and multilayer nitride coatings was presented in detail.* *Coating phase composition was obtained using Shimadzu XRD-6000 X-ray diffractometer at Cu-Кα radiation, voltage 30 kV, and current 20 μА. The failure pattern and surface morphology, grain size and coating thickness of single-layer and multi-layer coatings were examined using the field emission electron microscope Ultra 55 with EDAX microanalyzer. To determine metal concentration in the coatings, a local chemical analysis was carried out by EDAX microanalyzer.* *Electrochemical tests (polarization curves, electrochemical impedance spectroscopy (EIS)) of substrate (HG30, X165CrMoV12, and USt 37-2) with single layer and multilayer nitride coatings were performed in neutral chloride solutions (0.3% NaCl, 3% NaCl), and HG30 was tested also in 5% NaOH.* *The impedance spectrum was measured initially at the open-circuit potential in the frequency ω/2π range from 30 kHz to 0.003 Hz with Solartron 1255 frequency response analyzer and Solartron 1287 potentiostat (Solartron Analytical). Then the impedance was measured at anodic polarizations in the frequency range from 10 kHz to 0.01 Hz. The amplitude of the ac signal was 10 mV, and the duration of the current stabilization at each potential before impedance spectrum measurement was 10 min.*
Data format	*Raw, analyzed*
Experimental factors	*The deposition process data were optimized to obtain single-layer coatings with preset structure, phase and elemental composition, and a maximum protective effect in 5% NaOH or 3% NaCl. The data were obtained through assessing the data of grain size and coating thickness, phase and elemental composition of multilayer coatings on their protection effectiveness in both 5% NaOH and 3% NaCl.*
Experimental features	*The experiments were designed for the optimization of structure, phase and elemental composition of each layer and the multilayer coating as a whole.*
Data source location	*Perm National Research Polytechnic University, Perm, Russian Federation*
Data accessibility	*All data are presented in this paper.*
Related research article	*A.L. Kameneva, V.I. Kichigin, Corrosion, wear, and friction behavior of a number of multilayer two-, three- and multicomponent nitride coatings on different substrates, depending on the phase and elemental composition gradient//Applied Surface Science. 489 (2019) 165–174.*

**Table:** 

**Value of the Data** • These data could be helpful for deposition of single-layer and multilayer coatings based on two-, three- and multi-component nitrides. • The data obtained could be used to check corrosion behavior of single-layer coatings and their influence on the corrosion of different substrate in the 3% NaCl and 5% NaOH. • The data can be used to receive multilayer two-component nitride coatings with gradient of structure, phase and elemental composition and corrosion resistance both in 5% NaOH, and in 3% NaCl. • According to the data presented, it is possible to establish the optimal phase and elemental composition of nitride coatings with a maximum protective effect in the 3% NaCl and 5% NaOH.

## 1 Data

### 1.1 Deposition process characteristics of the single-layer coatings

Material of substrates, single-layer coatings and method of their deposition are shown in Table 1. The material and deposition method for sublayers and coatings were chosen the same. The data presented in this paper illustrate the optimization of the technological parameters of DC magnetron sputtering (MS), cathodic arc evaporation (CAE), pulsed magnetron sputtering (PMS), and combined methods (CAE + MS and CAE + PMS). The technological parameters include evaporator arc current (*I*_*arc*_), magnetron discharge power (*N*), bias voltage on the substrate (*U*_bias_), partial pressure of the gas mixture of nitrogen and argon (*P*), the nitrogen content in the gas mixture (N_2_) and the duration of the coating deposition (*T*_с_). The values of technological parameters of coating deposition, given in Table 2, were changed to establish the phase and element composition of the single-layer coatings with the maximum protective effect in 5% NaOH and 3% NaCl solutions. The optimum value of the technological parameters in Table 2 are highlighted in bold.

**Table 1:** Material of substrates, single-layer coatings and method of their deposition.

Coating no.	Substrate	Coating material	Deposition method
1	HG30	TiN	CAE
2			MS
3		ZrN	CAE
4			MS
5		(Ti,Zr)N	CAE
6			MS
7			CAE + MS
8	HG30 and X165CrMoV12	(Ti,Al)N	PMS
9		TiN	PMS
10	USt 37-2	(Ti,Zr,Al)N	CAE + MS

**Table 2:** Technological parameters of single-layer coating deposition.

Coating no.	*P*, Pa	*I*_*arc*_, A		*N*, kW			*U*_bias_, V	N_2_, %
		Ti	Zr	Ti	Zr	Al		
0	1.0	–	75	–	2.5	–	80, 200	Ar
00		75	–	2.5	–	–		
000		75	75	2.5	2.5	–		
1	0.8; **1.0**; 1.2	80	–		–	–	80	40
2		–	–	2.5	–	–	80, 150, **200**	100
3	0.8; **1.0**; 1.2	–	–	5.1; **5.5**; 6.8		–	60	90
4		–	80	–	–	–	80	40
5		–	–	–	1.7; 1.8; 1,9; 2.0;**2.5**	–	200	100
6		75	75	–	–	–	40, 60, **80**	30, **35**, 40
7		–	–	2.0	2.0		200	100
8	0.24; 0.26; **0.28**	80	–	2.0	2.0	–	40, **90**	50
9	0.28; **0.5**; 0.8	–	–	**7.0**, 9.0	–	7.0	**50**, 55	**12**, 19
10	1.0	90	–	–	2.1	1.6	70	40

### 1.2 Analysis of phase and elemental composition of single-layer nitride coatings obtained at different technological parameters

**Fig. 1:**
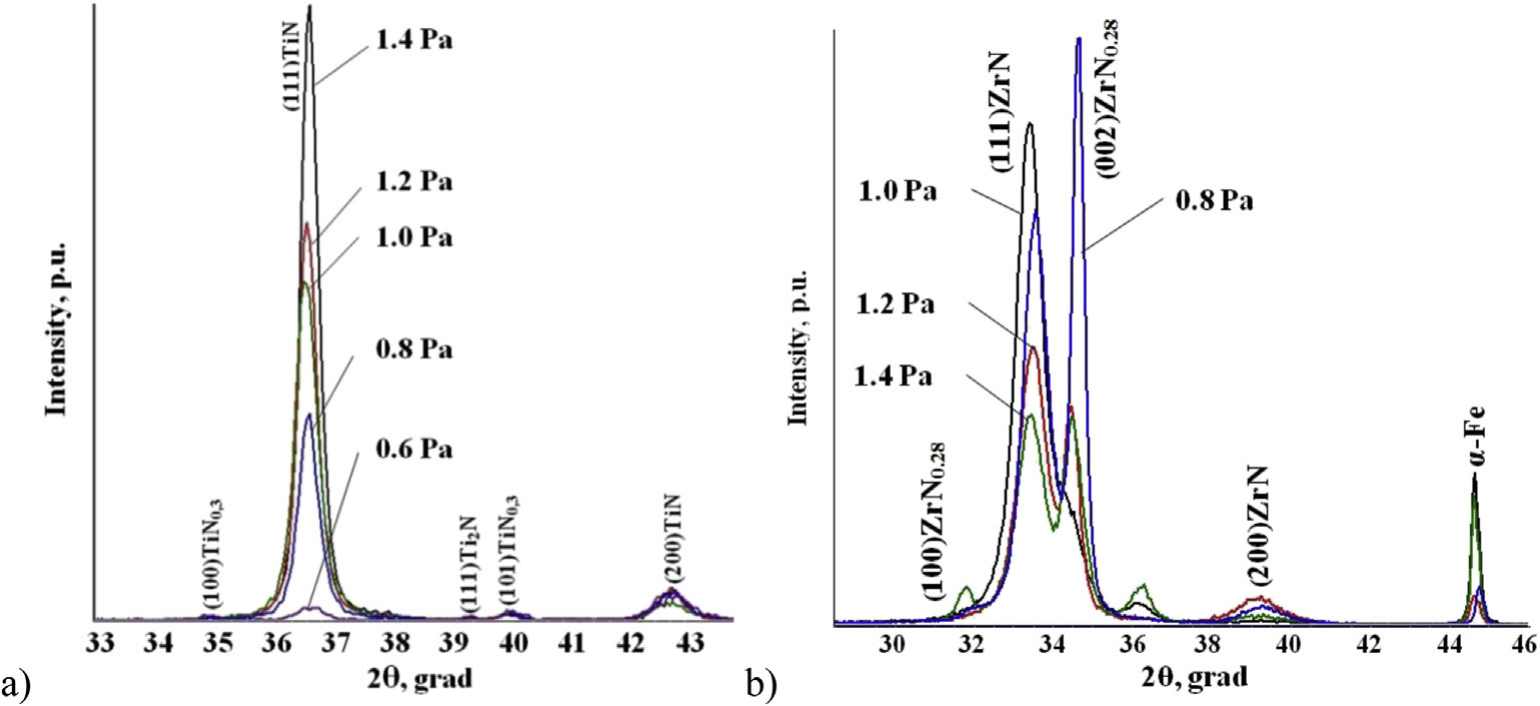
Combined fragments of X-ray diffractogram of coatings obtained by cathode-arc evaporation at various pressure of the gas mixture: TiN (a) and ZrN (b).

**Fig. 2:**
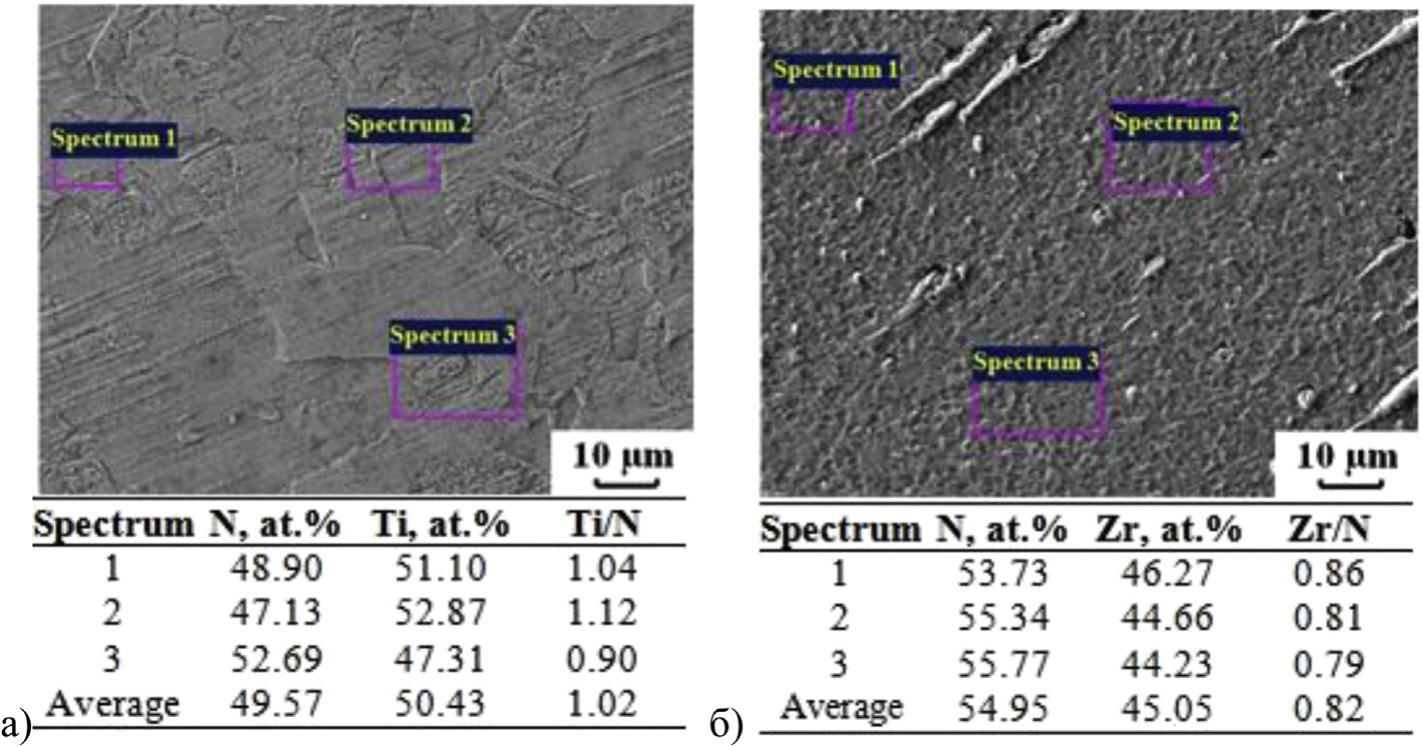
Elemental composition in different spectrums of coating: TiN (a) and ZrN (b).

Phase analysis data was plotted as combined fragments of X-ray diffractogram of coatings, obtained at different technological parameters. Fig. 1 illustrates the phase composition of single-layer TiN and ZrN coatings obtained by cathodic arc evaporation at the different gas mixture pressure. The phase composition of coatings 1–10 with maximum volume content of phases c-TiN, c-ZrN, δ-Zr_3_N_4_, c-TiZrN_2_, h-Ti_3_Al_2_N_2_ are shown in Table 3. The elemental composition was determined only for coatings which phase composition is demonstrated in Table 3. The average values of the elemental composition of the coatings, determined in three different spectrums, are shown in Table 3 and Fig. 2.

**Table 3:** Phase and elemental composition of single-layer coatings. Other phases are AlN, TiN_0.3_, ZrN_0.28_, V_Zr2N_, Ti_2_AlN.

Coating no.	*V* – Coating phase volume content (%)						Coating metal content (at.%)		
	TiN	ZrN	Zr_3_N_4_	TiZrN_2_	Ti_3_Al_2_N_2_	Other phases	Ti	Zr	Al
1	96.8	–	–	–	–	3.2	100	–	–
2	99.2	**–**	**–**	**–**	–	0.8	100	–	–
3	–	91.7	–	–	–	8.3	–	100	–
4	–	100	–	–	–	–	–	100	–
5	99.0	–	0.6	0.4	–	–	84.9	15.1	–
6	15.1	–	13.4	71.5	–	–	27.3	72.7	–
7	18.4	–	51.6	30.0	–	–	69.1	30.9	–
8	10.0	–	–	–	86.0	4.0	53.1	–	45.7
9	84.5	–	–	–	–	15.5	100	–	–
10	13.8	–	57.6		–	28.6	38.0	53.9	8.1

### 1.3 Optimization of the corrosion properties by phase and elemental composition, structure, grain size, and thickness of single-layer nitride coatings

**Fig. 3:**
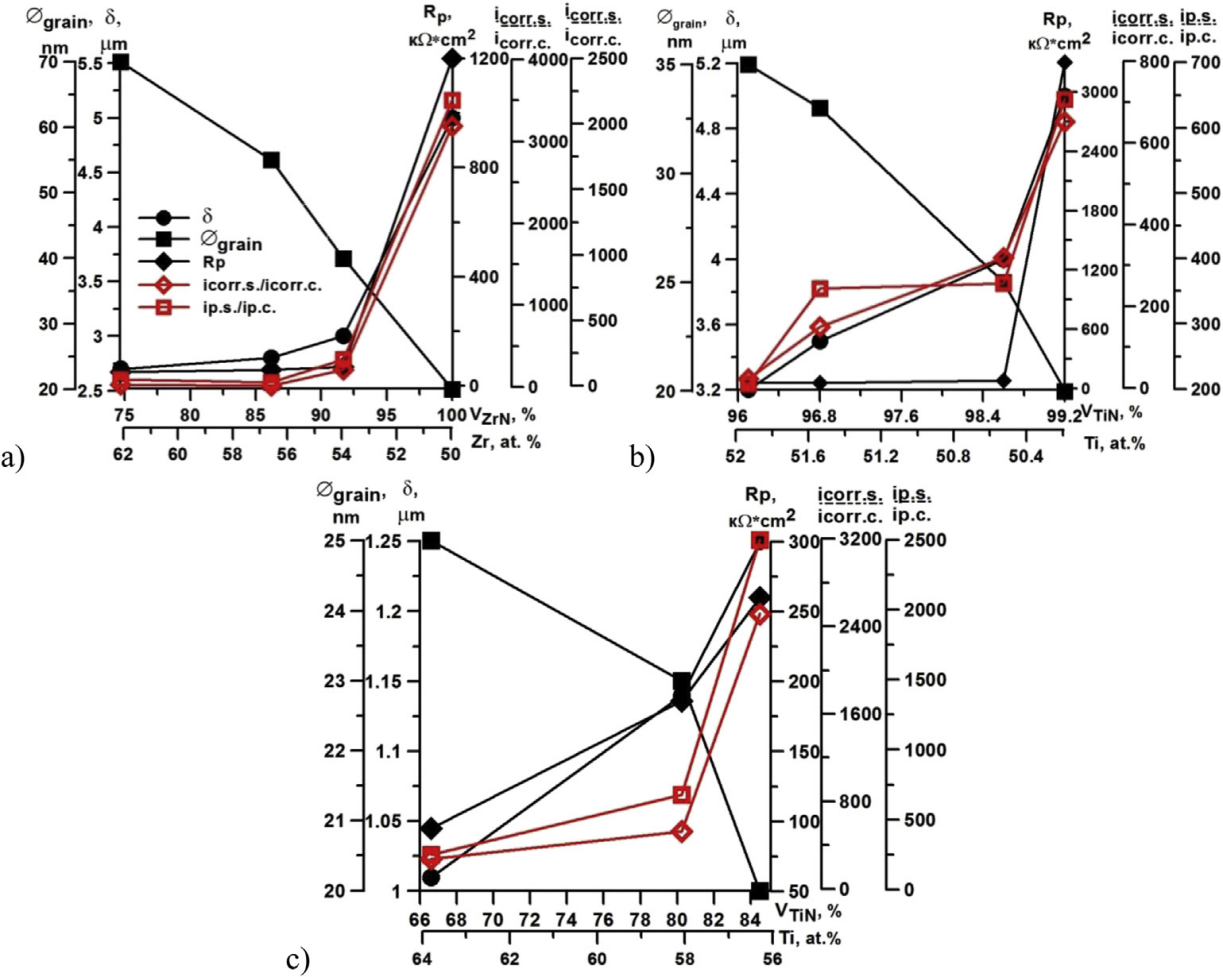
Dependence of coating efficiency in 5% NaOH and 3% NaCl on the phase and elemental composition of single-layer coatings ZrN CAE (a), TiN CAE (b), TiN PMS (c).

The coating deposition method was optimized by corrosion properties data (Table 4). Corrosion properties of the substrate and the single-layer coatings with optimal phase and elemental composition are presented in Table 4. Here E_corr_ is the corrosion potential, R_p_ is the polarization resistance, i_corr_ is the corrosion current density, i_p_ is the passive current density; subscripts s and c denote substrate and coating, respectively. The coatings based on c-TiN and c-ZrN phases have the maximum protection efficiency. The data on optimization of the volume and metal content, grain size and thickness of these coatings are presented in Fig. 3. The greatest value of Rp, icorr,s/icorr.c, and ip,s/ip,c always corresponds to the minimum grain size, maximum volume content of c-ZrN and c-TiN phases, optimum thickness and composition close to stoichiometric (Fig. 3).

**Table 4:** Corrosion properties of substrate and single-layer coating.

Substrate material/Coating no.		*T*_layer_, min	*δ*_max_, μm	Corrosion properties			
				(−)E_corr_, V	R_p_, kΩ·cm^2^	*i*_corr,s_ *i*_corr.c_	*i*_p,s_ *i*_p,c_
HG30	5% NaOH	–	–	0.36	27	–	–
	3% NaCl			0.07	8.5	–	–
X165CrMoV12	0.3% NaCl	–	–	0.27	1.9	–	–
USt 37-2	3% NaCl	–	–	0.42	2.4		
1	5% NaOH	30	5.0	0.12	30	22	362
2		45	4.0	0.10	3300	652	643
3		10, 12, 45	4.2	0.32	70	208	240
4		8, 30	5.5	0.14	1200	3190	2180
5		6	1.3	0.11	222	780	720
6		10	1.1	0.32	48	325	330
7		5, 8, 10	2.0	0.20	52	370	385
8	5% NaOH	80	3.3	0.40	20	10	8
	0.3% NaCl						
9	0.3% NaCl	80	4.0	0.14	150	43	–
	3% NaCl						
10	3% NaCl	30	5.0	0.01	62	25	–

The stoichiometric nanostructured coating ZrN with maximum volume content of the phase c-ZrN and zirconium concentration, optimum thickness of 5.5 μm, and grain size of 20 … 70 nm slows down the corrosion in 5% NaOH by over 3000 times, and the passive current – by 2000 times (Fig. 3a). The increasing of phase volume of h-ZrN_0.28_ up to 8% decreases the ratios *i*_corr,s_*/i*_corr,c_ and *i*_p,s_*/i*_p,c_ to 208 and 240 times, respectively.

Optimal phase composition of TiN coating is *V*_c-TiN_ = 99.2% and *V*_h-TiN0.3_ = 0.8%, optimum thickness of 5 μm, and grain size of ∼20 … 35 nm. The ratio *i*_corr,s_*/i*_corr,c_ for this coating is over 650, and the ratio *i*_p,s_*/i*_p,c_ is equal to 640. When increasing h-TiN_0.3_ phase content, the ratios *i*_corr,s_*/i*_corr,c_ and *i*_p,s_*/i*_p,c_ decrease to 20 and 360, respectively (Fig. 3b).

By values of ratios *i*_corr,s_*/i*_corr,c_ and *i*_p,s_*/i*_p,c_, single-layer coating TiN, deposited by PMS, has the best corrosion resistance in the 3% NaCl. This coating on a HG30 substrate with the preferred crystal orientation (111), a thickness of more than 1 μm, volume content of phases *V*_c-TiN_ = 84.5% and *V*_h-TiN0.3_ = 15.5%, and minimum grain size of ∼20 nm shows a higher propensity to anodic passivation in 3% NaCl and most effectively slows down the corrosion process. The ratios characterizing the coating protection efficiency are *i*_corr,s_/*i*_corr.c_ ≈ 2500, *i*_p,s_/*i*_p,c_ = 2500. Nanostructured denser coatings deposited by the PMS method are less sensitive to the coating thickness and concentration of low-nitrogen phase h-TiN_0.3_ (Fig. 3c).

**Fig. 4:**
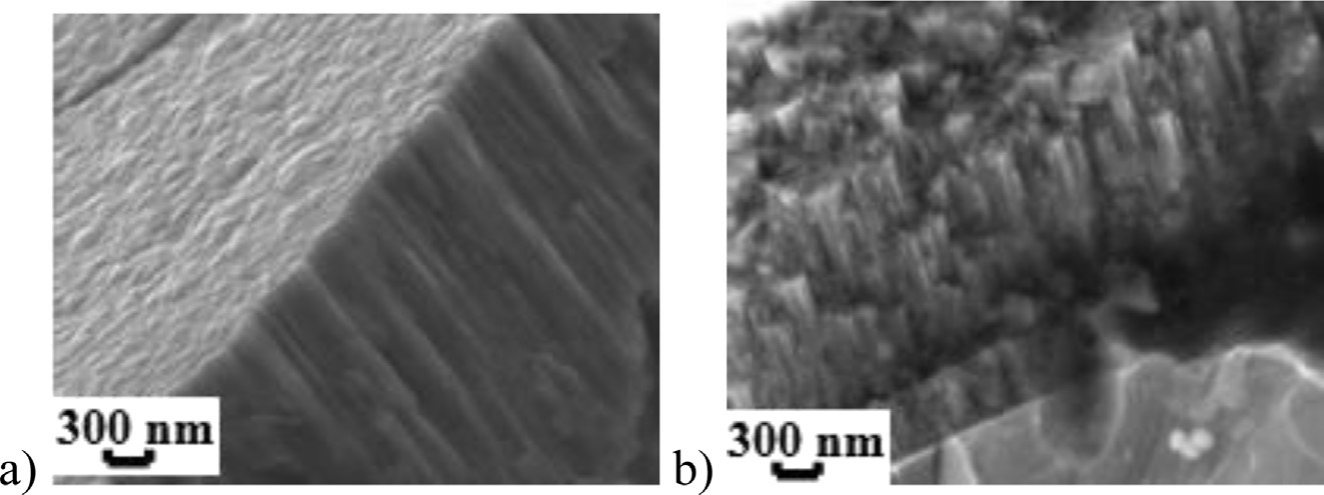
Nanostructured coatings on hard alloy HG30: single-layer (a), multi-layer (b).

The fracture pattern of single-layer coating and its surface morphology, obtained using an Ultra 55 field emission electron microscope, are shown in Fig. 4a.

### 1.4 Corrosion behavior of multilayer coatings with alternating layers and gradient structure, phase and elemental composition

Multilayer coatings (Table 5) were developed on the basis of single-layer nanostructured nitrides ZrN, TiN and (Ti,Zr)N with a maximum protective effect in the 5% NaOH and 3% NaCl. In particular, two-component nitrides ZrN and TiN with maximum resistance in the 5% NaOH and 3% NaCl, respectively, were used as alternating layers for multilayer coatings No. 11 and No. 12. The layer deposition methods for these coatings were reversed to obtain them with different structure, phase and elemental composition. The phase and elemental composition of multilayer coatings, and their corrosion properties are given in Table 6. The layers of multilayer coatings were deposited at optimal values of the process parameters. The thickness of the layers ranged from 100 nm to 500 nm with a total thickness of the multilayer coating ranging from 0.5 to 5.0 μm.

**Table 5:** Coating materials and deposition methods (substrate material – HG30, sublayer - TiN).

Coating No.	Coating material/Alternate layers	Deposition method	
		Ti	Zr
11	TiN–ZrN	MS	CAE
12	TiN–ZrN	CAE	MS
13	(Ti,Zr)N–Ti,Zr	CAE	CAE
14	ZrN–Zr-(Ti,Zr)N–Zr	MS	MS
15	TiN-(Ti,Zr)N	MS	CAE

**Table 6:** Corrosion potentials, polarization resistance, and corrosion and passive current density. ratios for multi-layer coatings. Coatings 11, 13–15 were tested in 5% NaOH, coating 12 - in 0.3% NaCl.

Coating No	*V* - Coating phase volume content (%)						Metal content in the coating (at.%)		Corrosion properties			
	TiN	ZrN	Zr_3_N_4_	TiZrN_2_	TiN_0.3_	ZrN_0.28_	Ti	Zr	(−)E_corr_, V	R_p_, kΩ·cm^2^	*i*_corr,s_ *i*_corr.c_	*i*_p,s_ *i*_p,c_
11	59.0	–	7.0	34.0	–	–	42.00	35	0.04	550	750	800
12	24.1	49.6	2.5	2.9	2.8	18.1	5.42	74.31	0.04	186	136	229
13	–	–	29.0	71.0	–	–	55.15	26.79	0.05	110	417	400
14	92.0	–	3.0	5.0	–	–	63.82	22.69	0.05	30	150	200
15	87.0	–	5.0	8.0	–	–	55.62	21.61	0.08	100	273	300

Based on the values of *E*_corr_, *R*_p_, *i*_corr,s_/*i*_corr,c_ and *i*_p,s_/*i*_p,c_, TiN–ZrN multilayer coatings (No. 11 and No. 12) have high corrosion resistance both in 5% NaOH, and in 3% NaCl. The corrosion inhibition effect *i*_corr,s_/*i*_corr,c_ and the surface passivation degree *i*_p,s_/*i*_p,c_ for these coatings in the 5% NaOH are 750 and 800 and in the 3% NaCl – 136 and 229, respectively. The fracture pattern of multilayer coatings, obtained using an Ultra 55 field emission electron microscope, are shown in Fig. 4b.

**Fig. 5:**
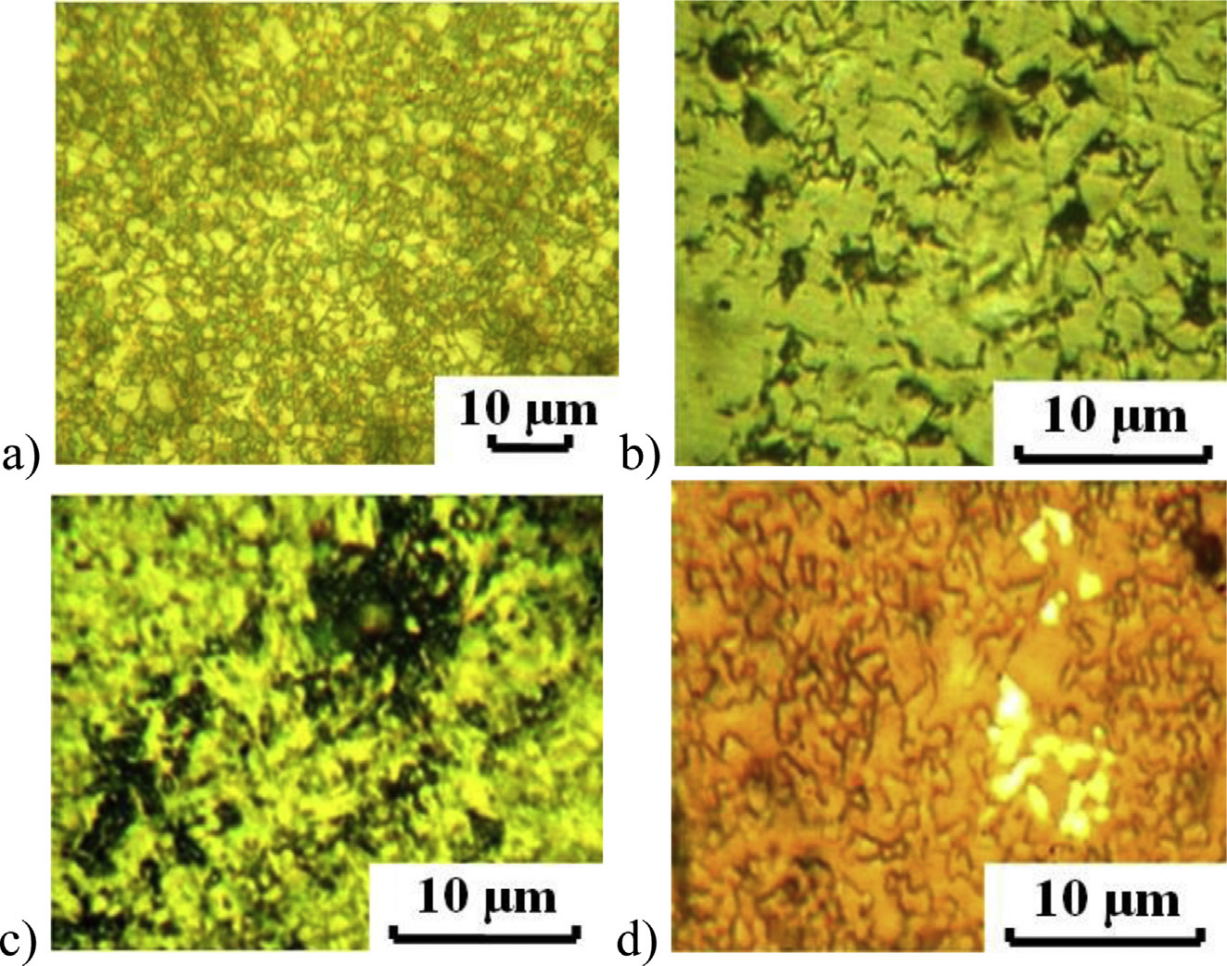
The surface morphology of hard alloy HG30 before electrochemical tests (a), HG30 after etching in a 5% NaOH solution (b), HG30 after anodic polarization in a 5% NaOH solution (c), HG30 with TiN–ZrN multilayer coating after anodic polarization of 5% NaOH solution (d).

**Fig. 6:**
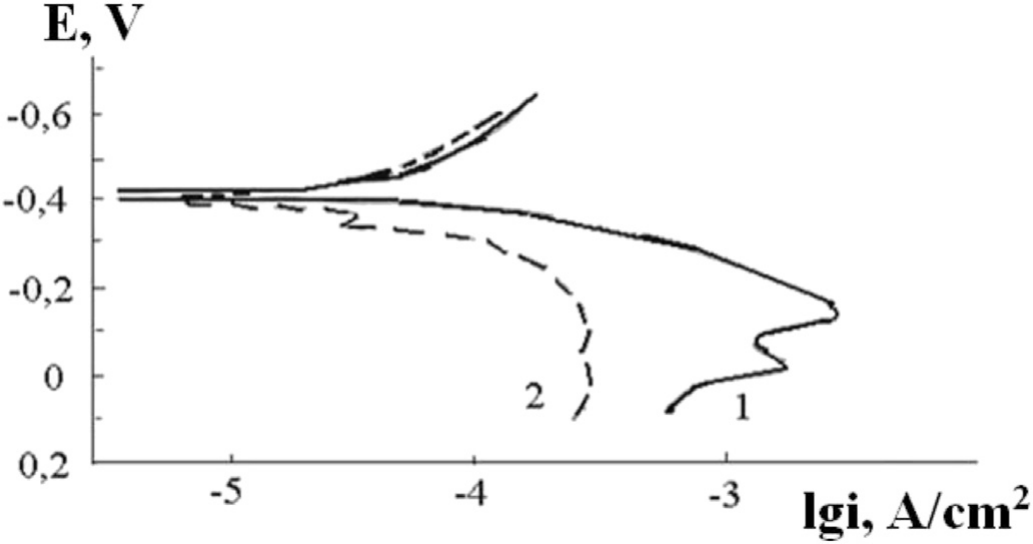
Anodic polarization curves in 3% NaCl solution: 1- hard alloy HG30 without coating, 2- HG30 with TiN–ZrN multilayer coating.

The surface morphology of hard alloy HG30 before electrochemical tests is illustrated at Fig. 5a. The surface morphology of hard alloy HG30 after the electrochemical tests in 5% NaOH solution is shown in Fig. 5b-d. After 5 minutes of etching in a 5% NaOH solution, the cobalt bond is slightly etched, the grain boundaries of tungsten carbide are exposed and crystallographic etching pits are revealed, the largest of which have a diameter of no more than 1.5 μm (Fig. 5b). After anodic dissolution in a 5% NaOH solution, crystallographic faceting of grains almost disappears on the VK8 surface, and dark etched areas are observed, which occupy a significant part of the surface of the test sample (Fig. 5c). After anodic dissolution in a 5% NaOH solution, sections of a golden multilayer coating are observed. No crystallographic pits were observed (Fig. 5d). Polarization curves of HG30 alloy in a 5% NaOH solution are given at Fig. 6. The dissolution rate at the potential of −0.2 V decreases by ∼18 times.

## 2 Experimental design, materials, and methods

### 2.1 Preparation of specimen with coatings

The hard alloy VK8 (HG30 – standard DIN), tool steel X12 M (D2 – ASTM or X165CrMoV12 – DIN), and low carbon steel St3 (1017 – ASTM and USt 37-2 – DIN) were used as substrates. Single-layer coatings with different structure, phase and elemental composition were formed on the substrate by different methods. Two-component TiN and ZrN, and three-component (Ti,Zr)N coatings were deposited both by DC magnetron sputtering (MS) and cathode arc evaporation (CAE). Two- and three-component coatings of TiN and (Ti,Al)N, and three-component and multi-component coatings of (Ti,Zr)N and (Ti,Zr,Al)N were formed by the pulsed magnetron sputtering (PMS) and combined method, respectively. In particular, a three-component layer (Ti,Zr)N was deposited during the sputtering of the Ti target with a magnetron sputter and the evaporation of the Zr cathode was carried out by an arc evaporator. In the process of forming a multicomponent layer (Ti,Zr,Al)N, two magnetron sputters with Zr and Al targets and one arc evaporator with a Ti cathode were used [[1], [2], [3], [4], [5], [6], [7], [8], [9], [10], [11], [12]]. The technological parameters were changed in the following ranges: evaporator arc current (I = 75–80 A), magnetron discharge power (N = 1.5–9.0 kW), bias voltage on the substrate (Ubias = 40–200 V), partial pressure of the gas mixture of nitrogen and argon (P = 0.24–1.4 Pa), the nitrogen content in the gas mixture (N2 = 12–100%) and the duration of the coating deposition (Tс = 5–45 min). In the deposition process one of the technological parameters was changed. Multilayer coatings were deposited within the optimal range of technological parameters. Automatic unit URM3.279.048 with two arc evaporators and four magnetron sputters and automatic unit of pulsed magnetron sputtering UNICOAT-600 were used for the deposition of coatings.

### 2.2 Phase and elemental analysis and SEM

Coating phase content was obtained using Shimadzu XRD-6000 X-ray diffractometer at Cu-Кα radiation, voltage 30 kV, and current 20 μА. Cubic TiN, ZrN, TiZrN_2_, Ti_3_AlN, hexagonal ZrN_0.28_, TiN_0.3_, Ti_2_AlN, Ti_3_Al_2_N_2_, and orthorhombic Zr_3_N_4_, Zr_2_N and Zr_3_AlN phases formed in the coatings are further referred to as c-TiN, c-ZrN, c-TiZrN_2_, c-Ti_3_AlN, h-ZrN_0.28_, h-TiN_0.3_, h-Ti_2_AlN, h-Ti_3_Al_2_N_2_, δ-Zr_3_N_4_, δ-Zr_2_N, and δ-Zr_3_AlN, respectively [[7], [8], [9]]. Phase changes in the formed coatings were estimated by phase volume content (*V*_TiZrN2_, *V*_TiN_, *V*_Zr3N4_ etc.). The failure pattern and surface morphology, grain size and coating thickness of single-layer and multi-layer coatings were examined using the field emission electron microscope Ultra 55 with EDAX microanalyzer. To determine metal concentration in the coatings, a local chemical analysis was carried out by EDAX microanalyzer.

### 2.3 Electrochemical tests (polarization curves, electrochemical impedance spectroscopy (EIS))

Electrochemical tests (polarization curves, electrochemical impedance spectroscopy (EIS)) were performed in neutral chloride solutions (0.3% NaCl, 3% NaCl), and samples of HG30 were tested also in 5% NaOH. The electrodes for electrochemical measurements were embedded in epoxy, leaving only one side of the sample in contact with the solution. Electrode surface was cleaned with ethanol and washed in the working solution. Polarization and impedance data on electrodes were measured at room temperature (22–24 °C) in a three-electrode cell in unstirred solutions without deaeration. The electrodes were inserted into a solution and the open-circuit potential was monitored until a steady-state potential was achieved. The impedance spectrum was measured initially at the open-circuit potential in the frequency *ω*/2*π* range from 30 kHz to 0.003 Hz with Solartron 1255 frequency response analyzer and Solartron 1287 potentiostat (Solartron Analytical). Then, the impedance was measured at anodic polarization in the frequency range from 10 kHz to 0.01 Hz. The amplitude of the ac signal was 10 mV, and the duration of the current stabilization at each potential before impedance spectrum measurement was 10 min. The CorrWare, ZPlot, CorrView and ZView software (Scribner Associates, Inc.) was used for the measurements and data processing. The electrode potentials *E* are reported with respect to the standard hydrogen electrode. The corrosion potential (*E*_corr_), the polarization resistance (*R*_p_), the corrosion current densities ratio for uncoated and coated substrate (*i*_corr,s_/*i*_corr,c_ – corrosion inhibition effect) and that of passive current densities for the same samples (*i*_p,s_/*i*_p,c_ – coating surface passivation degree) were determined to characterize the corrosion protection efficiency of coatings. The polarization resistance was found from impedance data as the limit of the real part of impedance at *ω* → 0 minus the solution resistance [13]. The corrosion current densities (*i*_corr_) were determined by extrapolation of cathodic and anodic parts of polarization curves to the corrosion potential. The passive current densities (*i*_p_) were directly taken from the anodic polarization curves [1,[3], [4], [5],^7^,^11^].
